# Omics and Multiomics-Based Diagnostics for Invasive Candidiasis: Toward Precision Medicine

**DOI:** 10.1016/j.mcpro.2025.101463

**Published:** 2025-11-12

**Authors:** Aida Pitarch, Víctor Arribas, Concha Gil

**Affiliations:** Faculty of Pharmacy, Department of Microbiology and Parasitology, Complutense University of Madrid (UCM), Madrid, Spain

**Keywords:** *Candida* albicans, invasive candidiasis, diagnosis, panomics, translational medicine

## Abstract

Invasive candidiasis (IC) is a serious, life-threatening, and costly fungal infection if not diagnosed early and treated appropriately. However, this healthcare-associated mycosis caused by *Candida* spp. is difficult to diagnose because of its nonspecific clinical signs and symptoms, and the lack of early and accurate detection methods. IC is also difficult to treat due to its late diagnosis, the limited antifungal arsenal, and the rapid emergence and spread of (multi)drug-resistant *Candida* strains. Therefore, early and accurate innovative methods for species and resistance identification in IC (candidemia and deep-seated candidiasis) are urgently needed to initiate timely and appropriate antifungal therapy, and reduce its high morbidity, mortality, and health care costs in hospitalized patients (especially, severely immunocompromised or critically ill patients). The availability of the complete genome sequences of the most clinically relevant *Candida* species coupled with recent advances in high-throughput omics technologies have spurred an unprecedented era in the discovery and development of IC diagnostics at different levels of molecular complexity. Here, we review the contribution of current and emerging omics technologies, including genomics, transcriptomics, proteomics, peptidomics, metabolomics, lipidomics, glycomics, immunomics (immunoproteomics, immunopeptidomics, and immunoglycomics), imiomics (imaging-omics), and microbiomics (metagenomics, metatranscriptomics, metaproteomics, and metabonomics), to the process of biomarker development for early diagnosis, antifungal susceptibility, prognosis, follow-up, and therapeutic monitoring in IC. We highlight the potential of integrating multiple omic data (through integromics, multiomics, or panomics, together with systems biology and artificial intelligence) for the discovery of multidimensional biomarker signatures and computational algorithms for IC diagnosis. Finally, we discuss future challenges and prospects for their clinical implementation. These next-generation IC diagnostics promise to revolutionize medical practice by unraveling the complexity of biological systems at multiple levels. Furthermore, these could help clinicians make more precise and personalized clinical decisions through multiomics or panomics-based precision medicine approaches, rather than traditional one-size-fits-all approaches.

## Highlights

• Genomic and proteomic diagnostics for invasive candidiasis (IC) are in clinical use.
• Transcriptomic, metabolomic, lipidomic, and glycomic IC tests remain investigational.
• Peptidomic and imiomic studies in IC patients are still in their infancy.
• Immunomics and microbiomics add immune and microbiota interaction to IC diagnosis.
• Multiomics and artificial intelligence promise to transform precision IC diagnostics.

## Graphical Abstract

**Figure:**
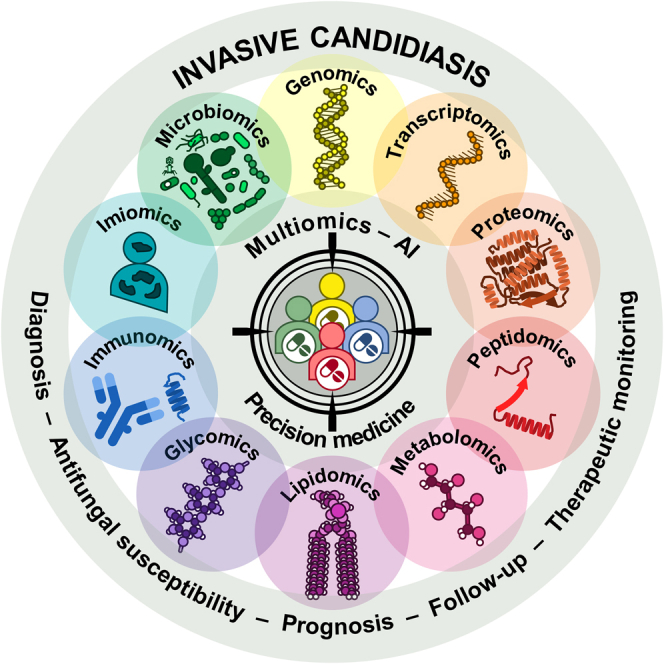

## In Brief

Early diagnosis of invasive candidiasis (IC) is difficult, but crucial for improving patient outcomes. Omics technologies (genomics, transcriptomics, proteomics, peptidomics, metabolomics, lipidomics, glycomics, immunomics, imiomics, and microbiomics) have identified new clinical biomarker candidates and signatures for IC at different levels of biology. However, most of them have not been translated into the clinic. The integration of this multilevel information into multidimensional biomarker signatures and computational algorithms through multiomics and artificial intelligence may pave the way toward precision medicine in IC.

Invasive candidiasis (IC) poses a persistent public health challenge with broad health, economic, and epidemiological implications worldwide (1, 2, 3, 4). Not only is IC the most common primary bloodstream infection and the seventh most frequent healthcare-associated infection (5), but it also remains a major infectious cause of morbidity and mortality in hospitalized patients with serious underlying diseases (such as cancer, transplant, intensive care, burn, post surgical, and neonatal patients) and carries significant health care and societal costs (2, 4, 6, 7, 8, 9, 10). Early diagnosis of IC (including candidemia and deep-seated candidiasis) is essential for a favorable clinical outcome, as its detection within the first 24 h is associated with a lower mortality risk (11, 12, 13). However, the lack of specific clinical signs and symptoms of invasive infection, as well as the time delay, inadequate accuracy, and insufficient standardization of currently available diagnostic methods hinder the early and accurate diagnosis of IC and, consequently, lead to delayed therapy and poor clinical outcomes (14, 15). In particular, blood cultures (the gold standard for IC) are not reliable for the diagnosis of deep-seated candidiasis in the absence of candidemia (11), and require an incubation period of 19 to 75 h for a positive result, and another 24 to 48 h for identification to species level by conventional assays (16, 17). Histopathological examination of normally sterile sites (the other reference standard of IC) has reduced diagnostic sensitivity, and may be very risky in critically ill or unstable patients and contraindicated in patients with severe thrombocytopenia or coagulopathies (14, 18).

IC is caused by some species of the genus *Candida*. Although *Candida albicans* is still the most common causative agent of IC, several non-albicans species (predominantly, *Candida parapsilosis*, *Candida glabrata*/*Nakaseomyces glabrata* (19), *Candida tropicalis*, *Candida krusei*/*Pichia kudriavzevii* (20), and, recently in some areas, *Candida auris*/*Candidozyma auris* (21)) have emerged as important etiological agents of IC over the last decades (22, 23, 24, 25). This is the result, at least in part, of the extensive use of first-line antifungals as prophylactic, pre-emptive, or therapeutic agents. These fungal species colonize the skin and mucosal surfaces of the human body, and are part of the oropharyngeal, gastrointestinal, and vaginal microbiota of most healthy individuals (26, 27, 28). In severely immunocompromised and critically ill patients, these commensal fungi can, however, turn into opportunistic pathogens and cause IC (6, 29). In 2022, the World Health Organization classified these major causative agents of IC as critical (*C. albicans* and *C. auris*/*C. auris*), high (*C. glabrata*/*N. glabrata*, *C. tropicalis*, and *C. parapsilosis*) or medium (*C. krusei*/*P. kudriavzevii*) priority fungi to guide research, development and public health measures due to their antifungal resistance, mortality, diagnostic and treatment options, incidence, complications, and sequelae (30). World Health Organization also warned that antifungal resistance currently represents one of the most serious threats to global public health, and must be addressed with the highest priority. Unfortunately, the recent emergence and rapid spread of antifungal drug-resistant or multidrug-resistant clinical isolates of several *Candida* species (especially *C. auris*/*C. auris*, *C. glabrata*/*N. glabrata* and *C. parapsilosis*) among at-risk patients are jeopardizing the scarcity of therapeutic options to combat IC (*i.e*., azoles, polyenes, and echinocandins), making its successful treatment even more difficult in many cases (1, 2, 31, 32, 33). Because of the different antifungal susceptibilities of *Candida* species and the limited arsenal of antifungals with clinical utility to counteract drug resistance and treat IC, there is an urgent need for new, early, and accurate methods not only for species-specific identification, but also for the detection of their antifungal resistance patterns. These could therefore facilitate the diagnostic and therapeutic decision-making process for IC, as well as reduce selective pressure for the evolution of antifungal (multi)drug resistance among *Candida* clinical isolates (34, 35, 36).

**Fig. 1:**
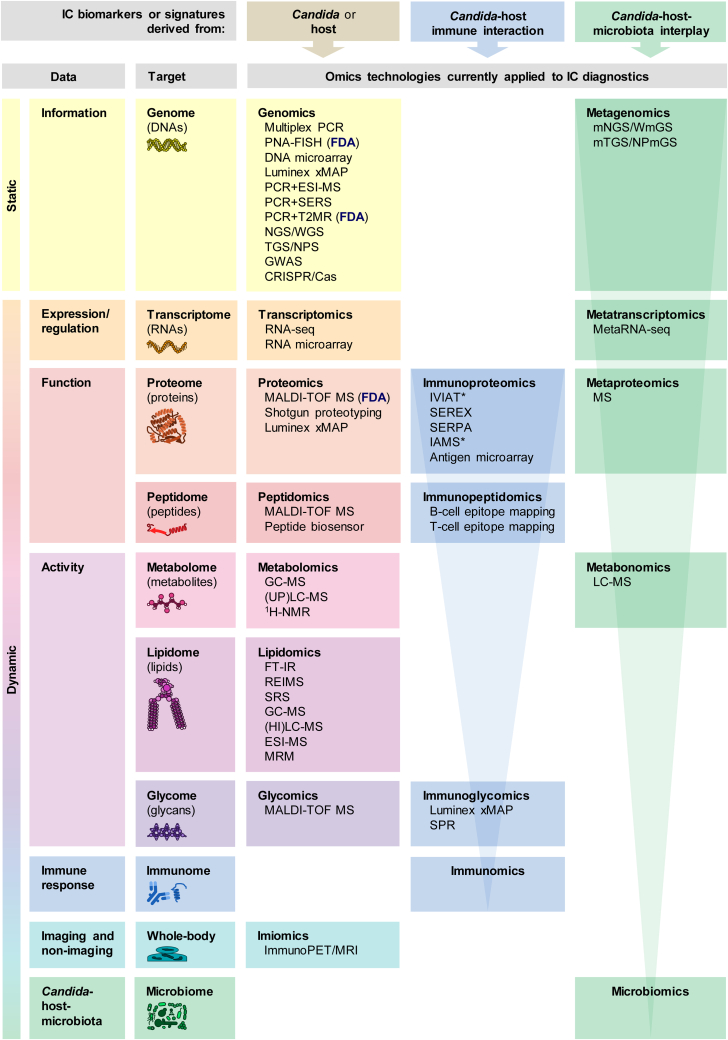
**Overview of the different omics approaches currently applied to IC diagnosis.** Methods approved by the FDA for IC diagnosis are indicated. *Asterisks* show technologies that have been used for the detection of *Candida* colonization or infection forms other than IC. See text for further details. ^1^H-NMR, proton nuclear magnetic resonance; Cas, CRISPR-associated protein; CRISPR, clustered regularly interspaced short palindromic repeats; ESI, electrospray ionization; FDA, Food and Drug Administration; FT-IR, Fourier transform infrared; GC, gas chromatography; GWAS, genome-wide association study; HILC, hydrophilic interaction liquid chromatography; IAMS, immunoaffinity mass spectrometry; IC, invasive candidiasis; IVIAT, *in vivo* induced antigen technology; LC, liquid chromatography; MALDI-TOF, matrix-assisted laser desorption/ionization time-of-flight; mNGS, metagenomic NGS; metaRNA-seq, meta-analysis of RNA-seq; MRI, magnetic resonance imaging; MRM, multiple reaction monitoring; MS, mass spectrometry; NGS, next-generation sequencing; NPS, nanopore sequencing; NPmGS, nanopore-based metagenomic sequencing; PCR, polymerase chain reaction; PET, positron emission tomography; PNA-FISH, peptide nucleic acid-fluorescent *in situ* hybridization; REIMS, rapid evaporative ionization mass spectrometry; RNA-seq, RNA sequencing; SEREX, serological analysis of recombinant complementary DNA expression libraries; SERPA, serological proteome analysis; SERS, surface enhanced Raman scattering; SPR, surface plasmon resonance; SRS, single-cell stimulated Raman scattering; T2MR, T2 magnetic resonance; TGS, third-generation sequencing; UPLC, ultra-performance liquid chromatography; WGS, whole genome sequencing; WmGS, whole metagenome sequencing; xMAP, multi-analyte profiling (x, analyte).

This relentless public health problem has motivated researchers to innovate and develop alternative technological solutions that allow clinicians to reach an earlier and more accurate diagnosis of IC and, as a result, minimize the clinical impact and economic burden of this life-threatening and costly fungal infection. Recent advances in high-throughput omics technologies are providing new opportunities for the rapid discovery of next-generation biomarker candidates for early diagnosis, prognosis, follow-up, and therapeutic monitoring of many diseases (including IC) with the aim of achieving personalization of health care (i.e., precision medicine or personalized medicine) (37, 38, 39). In this review, we provide an overview of how omics technologies (such as genomics, transcriptomics, proteomics, peptidomics, metabolomics, lipidomics, glycomics, immunomics, imiomics, and microbiomics) are contributing to the process of biomarker development for IC diagnosis (Fig. 1). We also discuss how the integration of these multiple omic data (through integromics, multiomics, or panomics) could usher in a new era of precision medicine.

## Genomics

Genomics, which studies the genomes of (micro)organisms, has matured faster than other omics in medicine, and has had a greater impact on the clinical management of infectious diseases (40, 41). Access to the genomes (the complete set of DNA or genetic information inside an organism) of most human pathogens has had important biological and clinical implications from the release of the complete genome sequences of the first virus (the bacteriophage MS2 in 1976), followed by the first prokaryote (the bacterium *Haemophilus influenza* in 1995) and eukaryote (the fungus *Saccharomyces cerevisiae*, a yeast species related to *Candida*, in 1996) to the present day (42, 43, 44, 45). It has not only contributed to our understanding of the pathogenesis, genetic diversity, and evolution of microorganisms, but also to the management of infectious diseases (46). The availability of the entire genetic content of most pathogens (including *C. albicans* in 2004, and other *Candida* spp. in 2009 (47, 48)), together with recent technological advances in clinical genomics, have led to the development of a plethora of molecular methods based on the rational selection of genomic targets for the diagnosis of infectious diseases (46, 49). These methods, ranging from diverse polymerase chain reaction (PCR)-based approaches to next-generation sequencing (NGS) techniques (Fig. 1), have been widely applied to diagnose IC and make more accurate clinical decisions in IC patients (38). However, further trials are still needed to elucidate their usefulness in routine clinical practice.

Over the last decades, specific DNA patterns, including several *Candida* genes (e.g., *ACT1*, *CHS1*, *ERG11*, *HSP90*, and *SAP1-6*) and highly conserved multicopy broad-range panfungal gene sequences or regions (e.g., 5.8S, 18S, 28S, ITS1, or ITS2), were detected by different PCR-based approaches for rapid identification of *Candida* spp. in whole blood, plasma, serum, other sterile fluids, or tissues of IC patients (15, 38, 50). At present, various commercial multiplex and broad-spectrum PCR assays for the identification of *Candida* species and their antifungal resistance profiles in IC are being incorporated into the routine workflow of most clinical microbiology laboratories worldwide (38, 51, 52, 53). Some of them allow rapid identification of *Candida* species directly from clinical samples without the need for culture (38). Although PCR-based assays showed to be highly sensitive and specific for IC diagnosis in a meta-analysis of 54 studies (50), these are prone to false-negative and false-positive results, and lack standardization and clinical validation, making their widespread application difficult (6, 15, 29).

Several DNA hybridization methods have also been developed for the molecular diagnosis of IC in clinical microbiology laboratories (38, 52, 53, 54). The yeast traffic light peptide nucleic acid-fluorescent *in situ* hybridization (PNA-FISH) assay (AdvanDx) is a commercial molecular method approved by the Food and Drug Administration (FDA) for rapid identification of common *Candida* spp. from positive blood cultures (15, 54). This DNA hybridization assay uses multiple fluorescent PNA probes targeting specific rRNA gene sequences of *C. albicans*, *C. parapsilosis*, *C. glabrata*/*N. glabrata*, *C. tropicalis*, and *C. krusei*/*P. kudriavzevii*. Although the PNA-FISH assay significantly reduces species-specific identification time (from 24-48 h to 30–90 min), it relies on positive blood cultures (which require 19–75 h for fungal growth, and can remain negative in up to 50% of IC cases), and misdiagnoses IC caused by other *Candida* spp. (11, 16, 54, 55). DNA microarrays are an alternative hybridization method that utilizes multiple probes (specific oligonucleotides or PCR products attached to a solid support) for the simultaneous detection and identification of panels of pathogens and antimicrobial resistance (56). This high-throughput multiplex detection approach was used to identify *Candida* species in clinical samples (mostly whole blood) and determine their antifungal susceptibility patterns (38, 57, 58, 59, 60, 61). Most of these DNA chips for IC diagnosis are still in the research phase. However, the Prove-it Sepsis assay (Mobidiag) is a commercially available broad-spectrum PCR-coupled microarray for easy, rapid, and accurate identification of major sepsis-causing agents (including *Candida* spp.) from positive blood cultures (60). The main advantages of DNA microarrays are their multiplexing, sensitivity, flexibility, and miniaturized size, while their most significant drawbacks are their technical requirements, lengthy optimization process, and cross-hybridization, among others (56, 62).

Other high-throughput technologies have been combined with DNA amplification techniques for IC diagnosis (38, 63, 64, 65, 66). Specifically, the Luminex multi-analyte profiling (xMAP) platform was applied to the molecular identification of *Candida* spp. and their antifungal resistance profiles in a rapid, accurate, and high-throughput manner (63, 67, 68, 69). Luminex xMAP technology utilizes labeled beads or microspheres for simultaneous measurement of multiple targets (in this case, genomic targets from multiplex PCR amplifications) within a single sample in a single reaction (70, 71). The combination of electrospray ionization (ESI) mass spectrometry (MS) with broad-range PCR proved to be a promising and accurate alternative for IC detection, but its complexity has hindered its clinical application to routine practice (38, 64). Surface-enhanced Raman scattering is a spectroscopic technique that, coupled to broad-range PCR, demonstrated high multiplexing capability and analytical sensitivity for the detection of *Candida* spp. in blood within 6 h (38, 65). However, the commercially available PCR-surface-enhanced Raman scattering assay (the RenDx Fungiplex assay (Renishaw Diagnostics Ltd)) lacks clinical evaluation in large patient cohorts (65). Notably, the T2*Candida* panel (T2Biosystems), which combines T2 magnetic resonance (T2MR) with multiplex PCR, is an FDA-approved commercial nanosystem for IC diagnosis that has minimized the time to initiation and use of antifungals among IC patients (66, 72). This allows the rapid and accurate detection of amplified DNA (hybridized to superparamagnetic nanoparticles) of five clinically relevant *Candida* species (*C. albicans*, *C. parapsilosis*, *C. glabrata*/*N. glabrata*, *C. tropicalis* and *C. krusei*/*P. kudriavzevii*) (15, 73, 74). This nanoassay is not dependent on positive blood cultures, and can detect *Candida* spp. at concentrations of 1 to 3 colony-forming units per milliliter (CFU/ml) in whole blood samples within 3 to 4 h. However, the T2*Candida* panel is associated with high costs, relies on the prevalence of IC in each clinical setting, and lead to false-negative results in patients with IC caused by other *Candida* species (16, 66, 73, 75).

As a result of improvements in sequencing speed and costs, multigene NGS is now helping clinicians detect IC when other diagnostic methods fail, and adjust antifungal therapy accordingly (76). Whole genome sequencing with NGS technology has enabled the identification of *Candida* species in the ascitic fluid of IC patients in the intensive care unit (ICU) (77), as well as the description of IC outbreaks caused by *C. albicans* and *C. parapsilosis* in neonates (78), *C. auris*/*C. auris* in the ICU (79), or multidrug-resistant *C. parapsilosis* isolates in hospital settings (80). Whole genome sequencing has also been useful in predicting antifungal (anidulafungin and fluconazole) resistance in *C. tropicalis* and *C. glabrata*/*N. glabrata* clinical isolates, which could help tailor treatment in IC patients (81, 82). Because this culture-independent, high-throughput, and precise technology may overcome some of the limitations of currently available methods for IC diagnosis, it is reasonable to expect that NGS could become a routine diagnostic technique in clinical microbiology laboratory in the future. However, clinical NGS, so-called short-read sequencing, is associated with high cost and long turnaround time (38, 83), and requires further standardization and validation studies in larger cohorts before its clinical usefulness can be established. These drawbacks have paved the way for the development of third-generation sequencing technologies, such as nanopore sequencing, which produce longer reads, resulting in reduced costs and turnaround time, real-time sequencing, and easier handling of the procedure (83, 84). In particular, nanopore sequencing has showed to be a sensitive and portable tool for rapid identification of *Candida* species in candidemia patients (85, 86), and of single nucleotide polymorphisms (SNPs) associated with antifungal resistance in clinical isolates of *Candida* spp. from blood cultures (87, 88). Despite its promising potential for the clinical management of IC, further research is needed for the routine clinical integration of this third-generation sequencing technology into the diagnosis of IC (86).

Genome-wide association study (GWAS) is an emerging approach used to identify associations between genetic variants (such as SNPs) and a specific trait or disease across many genomes (89). This genomic tool revealed SNPs in human genes related to host immune response that confer susceptibility to candidemia (90, 91, 92). Notably, a multicenter GWAS using a microarray of 118,989 SNPs at 186 loci associated with immune-mediated diseases uncovered three genetic risk factors for candidemia (SNPs in the *CD58*, *LCE4A-C1orf68*, and *TAGAP* loci), which were functionally validated by transcriptomic, pathway, and immunological analyses (90). GWAS also identified SNPs in *Candida* genes related to antifungal drug targets (*ERG11*, *FKS1*, and *FKS2*), drug efflux pumps (*CDR1*, *FLR1*, and *PDR1*), drug uptake permeases (*FCY2*), and adhesins (*EPA1-3*, *EPA6*, *EPA8*, *EPA9*, *EPA11*, *EPA12*, *EPA15*, *PWP1*, and *PWP2*), among others (93, 94, 95, 96, 97). These SNPs were associated with the risk of resistance or nonsusceptibility of *Candida* isolates to antifungal drugs, including azoles (fluconazole, itraconazole, voriconazole, and posaconazole), polyenes (amphotericin B), echinocandins (caspofungin, micafungin, and anidulafungin), and antimetabolites (5-flucytosine, and 5-fluorocytosine), in IC patients. These genetic biomarkers associated with increased susceptibility to candidemia (genetic variants in human populations) or with reduced susceptibility of *Candida* isolates to antifungal agents (genetic variants in *Candida* isolates) have the potential to be used as a risk prediction tool to guide diagnostic and therapeutic decision-making in IC. However, they should be further developed, validated, and refined for application in clinical settings.

In recent years, clustered regularly interspaced short palindromic repeats (CRISPR) and their CRISPR-associated (Cas) proteins, termed CRISPR/Cas systems, have emerged as a revolutionary diagnostic tool for fast, inexpensive, simple, affordable, ultrasensitive, and point-of-care (POC) detection of molecular targets (98, 99). CRISPR/Cas systems are key players in prokaryotic adaptive immune mechanisms that assist in recognizing and cleaving foreign nucleic acids (74). Several CRISPR/Cas9 and Cas12a-based approaches have been designed to provide rapid, accurate, cost-effective, and POC diagnosis for IC in routine clinical care (100, 101, 102, 103, 104), and to identify antifungal resistance patterns in clinical isolates of *C. parapsilosis*, *C. glabrata*/*N. glabrata*, or *C. auris*/*C. auris* recovered from IC patients (105, 106, 107, 108, 109). These promising genome editing tools integrated into ultrasensitive, easy-to-use biosensors have potential for use in POC and resource-limited settings (110), opening up a new generation of IC diagnostics.

## Transcriptomics

As genes are regulated under specific internal and external inputs in health and disease, the study of the transcriptome can be crucial for inferring how and which genes and pathways are actively expressed, connected, or altered by a disease (i.e., the link between genotype and phenotype), as well as for guiding diagnostic, prophylactic, and treatment decisions (111, 112). The transcriptome is the complete set of all RNA molecules expressed from genes in a cell, tissue, or organism at a given time, location and condition (111). Modern transcriptomic techniques, such as whole-transcript microarrays, RNA sequencing, NanoString, and quantitative reverse transcriptase PCR, have been widely used to profile the transcriptome of a biological system, and discover biomarkers and signatures for diagnosis, prognosis, follow-up, and therapeutic monitoring of various diseases (including infectious diseases) in biomedical research (113, 114, 115, 116). Despite recent technological advances, transcriptomic studies in IC patients are still in their early stages (Fig. 1).

Several studies based on transcript microarrays or RNA-sequencing highlighted that *Candida* triggered a specific host transcriptional response that accurately discriminated candidemia from bacteremia, or even viremia, in experimental animal models and in hospitalized patients (117, 118, 119). Most of the genes represented in these host transcriptomic signatures were associated with host defense against *Candida* and other pathogens (including *CXCL2*, *CXCL3*, *IL6*, *IL10*, *IRG1*, and *SOCS3*, among others) and heme biosynthesis, and were proposed as biomarker candidates for IC diagnosis. Furthermore, distinct blood RNA gene expression signatures validated in an independent cohort of mice could also differentiate between early and late IC, and predict progressive infection (117). These findings support the potential of host transcriptomics to uncover diagnostic and immune fingerprints of IC (in line with other infectious diseases (116, 120)). However, these signatures and biomarker candidates require clinical evaluation in larger cohorts of patients, and further development before clinical application can be achieved.

Transcriptome profiling also proved to be a promising tool for rapid and accurate identification of *Candida* species directly from blood cultures (121), antifungal susceptibility testing in IC (122), follow-up of IC (123, 124), monitoring of response to antifungal therapy in IC (125), and the discovery of potential therapeutic candidates against IC (126). More specifically, transcriptomics combined with phenotypic profiling revealed distinct genetic signatures that could differentiate between echinocandin-resistant and echinocandin-susceptible *C. auris*/*C. auris* strains (122). SeptiCyte RAPID (Immunexpress), a commercial, fully automated, rapid, host-response assay for detection of infection-related inflammation (sepsis) based on transcriptomics (quantification of the RNA transcripts, *PLA2G7* and *PLAC8*), was able to predict persistent candidemia (123). In silico genome-wide analysis identified a large panel of long noncoding RNAs that could serve as potential biomarkers to monitor candidemia caused by *C. auris*/*C. auris*, of which some were expressed from intergenic regions of genes involved in cell wall biogenesis, transport, and metabolism (124). In a murine model, two microRNAs (miR-204 and miR-211) associated with a protective effect on IC (related to kidney dysfunction) were shown to be useful prognostic biomarkers and therapeutic targets for IC (126). Although much work remains to be done, transcriptomics has uncovered several IC-related biomarker candidates and signatures that could enable the development of new diagnostic, prophylactic and therapeutic interventions for IC patients in the future.

## Proteomics

In recent decades, there has been increasing interest in analyzing the proteome (the entire set of proteins expressed by a cell, tissue, or organism at a given time, location, and condition) in biomedical research (127). The study of these functional biomolecules directly implicated in most physiological and pathological processes of living cells can provide relevant information on the biological processes and molecular functions underlying host-pathogen interaction and disease pathogenesis, and contribute to the identification of clinical biomarkers and therapeutic targets for infectious diseases (128, 129, 130). MS, protein microarrays, and targeted proteomics (e.g., multiple, selected, or parallel reaction monitoring; multiple reaction monitoring (MRM), selected reaction monitoring (SRM), or parallel reaction monitoring (PRM), respectively) are the most commonly used proteomic tools for the discovery, verification, and validation of clinical biomarkers and therapeutic targets for various diseases, including IC (Fig. 1) (131, 132).

Matrix-assisted laser desorption/ionization time-of-flight (MALDI-TOF) MS has become a rapid and accurate method for the identification of microorganisms (including *Candida* spp.) in routine practice in most clinical microbiology laboratories worldwide (133, 134). Their identification is based on their protein mass fingerprints (predominately of their abundant ribosomal proteins) (134). Several studies have demonstrated its potential not only for the identification of different *Candida* species, but also for the detection of antifungal resistance (135, 136, 137, 138, 139). In fact, two MALDI-TOF MS platforms, MALDI Biotyper (Bruker Daltonik GmbH) and VITEK MS (bioMérieux), were approved by the FDA for IC diagnosis (15). Although identification of *Candida* spp. using these platforms is rapid (10–30 min from positive blood culture bottles), cost-effective (low reagent and labor costs), accurate and species-level, it is dependent on positive blood cultures (see above), spectral database limitations, and elevated instrument cost (15, 140, 141).

In light of these limitations, other proteomic strategies based on targeted proteomics and Luminex xMAP technology have been developed to detect IC in high-risk patients (142, 143, 144, 145, 146, 147, 148). Using species-unique (proteotypic) peptides, shotgun proteotyping showed higher analytical sensitivity and diagnostic accuracy than MALDI-TOF MS and required a shorter incubation time before identification of *C. albicans* (142). Human cytokine patterns assessed in blood by Luminex xMAP technology were able to discriminate infants with fungal sepsis from those with bacterial sepsis or without sepsis, and revealed several biomarker candidates, such as interferon-γ, interleukin 10 (IL-10), interleukin 18 (IL-18), transforming growth factor-β, and tumor necrosis factor-α, for the diagnosis of candidemia in infants with sepsis (144). Other cytokine and chemokine biomarkers, including macrophage inflammatory protein-1β, interleukin 17A (IL-17A), and kynurenine, to name but a few, were also proposed for the detection of candidemia using Luminex xMAP technology, but their clinical usefulness remains controversial (145, 146, 147, 148, 149). Indeed, these proteomic assays are still in the research phase, and have not yet been standardized and validated in large independent cohorts of IC patients for routine clinical use.

## Peptidomics

Peptidomics is an emerging subfield of proteomics that focuses on the peptidome (the entire repertoire of natural or endogenous peptides within a biological sample at a given time, location, and condition) (150). Peptides are chains of 2 to 100 amino acids (with a molecular mass ranging from 200 Da to 10 kDa) that occur naturally in all organisms and are involved in various physiological and pathological processes, such as defense response (e.g., antimicrobial peptides), microbial virulence, and infection (e.g., peptide toxins, like candidalysin), stress regulation, growth regulation, cell-cell communication, or signal transduction, to name but a few (151, 152, 153). Recent advances in liquid chromatography (LC), MS, and bioinformatic analysis have facilitated the characterization of the peptidome of a (micro)organism, and highlighted its great potential to better understand disease pathogenesis, as well as to provide valuable candidates for the development of novel drugs, vaccines, and diagnostic tests (154, 155, 156). Despite their varied biomedical applications, peptidomic studies applied to IC diagnosis remain very limited (Fig. 1).

In a preliminary MALDI-TOF MS study, serum peptide profiles in a murine model of candidemia uncovered a combination of six specific mass-to-charge (*m/z*) peaks that could discriminate between candidemia and healthy mice with a diagnostic accuracy of 80% (157). These candidates could serve as a basis for the design of a rapid diagnostic method for IC. However, the identity of these potential peptide biomarkers and their diagnostic value in cohorts of patients at risk for IC remains to be determined.

A modular, single-component yeast biosensor integrating G protein–coupled receptors with a visible lycopene readout was designed for the detection of multiple naturally secreted fungal mating peptides (peptide pheromones) (158). This yeast biosensor proved to be a simple and sensitive assay to differentiate among *C. albicans*, *C. glabrata*/*N. glabrata*, and other clinically relevant pathogenic fungi. This assay was further optimized into a one-step dipstick prototype, resulting in a rapid, reliable, low-cost, low-tech method for specific detection of fungal species in blood, serum, or urine samples. This prototype could help guide diagnostic and therapeutic decisions, but lacks clinical evaluation.

## Metabolomics

Metabolites are the end products of cellular functions and are closely related to the phenotype of an organism. These low molecular weight (<1500 Da) biomolecules (such as nucleotides, amino acids, lipids, and sugars) are being studied to reveal alterations in metabolic pathways and cellular biochemical processes underlying host–pathogen interaction, and to identify clinical biomarkers associated with infectious diseases beyond classical technologies (159, 160, 161). Several analytical tools have been developed to characterize and quantify the metabolome (the complete set of metabolites within a biological specimen at a specific time, location, and condition), including nuclear magnetic resonance (NMR) spectroscopy, gas chromatography (GC) and LC combined with MS, Raman spectroscopy, and Fourier transform infrared (FT-IR) spectroscopy (159, 162). NMR and MS are the most widely used techniques for the detection of clinical biomarkers. NMR is more reproducible but less sensitive, and has a smaller dynamic range than MS. Although several applications of metabolomics in biomedical research have been described (162, 163, 164), its potential for searching for IC-related metabolic biomarkers and signatures, as well as for improving IC diagnosis, remains largely untapped (Fig. 1).

The first *Candida* metabolite biomarker discovered to diagnose IC was D-arabinitol (165). Various studies have evaluated its diagnostic utility in serum and urine samples from patients with IC caused by *Candida* species other than *C. glabrata*/*N. glabrata* and *C. krusei*/*P. kudriavzevii* (because these two species do not produce it) using GC-MS, GC-LC-MS, or automated enzymatic fluorometric assays (165, 166, 167, 168, 169, 170, 171). Interestingly, detection of the ratios of D-arabinitol to L-arabinitol (absent in fungi), and D-arabinitol to creatinine (a marker of renal dysfunction) made it possible to rule out false-positive results associated with the presence of D- and L-arabinitol isomers in humans and their increase in patients with renal dysfunction (due to their renal clearance), respectively (15, 170, 172, 173). Although the measurement of D-arabinitol has long been considered promising, this nonculture-based diagnostic test can lead to false-negative results in patients with IC caused by non-D-arabinitol-producing *Candida* species, and requires further standardization and validation (15, 169).

In a pilot GC-MS work, metabolome profiles from urine samples obtained before, at the time of, and after clinical diagnosis could differentiate between one preterm neonate with IC caused by *C. parapsilosis* and 13 healthy preterm controls (174). Some amino acids (*N*-glycine, L-threonine, and D-serine) and carbohydrates (D-glucose and maltose) with increased levels in IC, as well as organic acids (citric acid) and fatty acids (hexadecanoic acid and octadecanoic acid) with reduced concentrations in IC, were proposed as biomarker candidates for diagnosing IC and monitoring the efficacy of antifungal therapy in IC patients. However, these candidates have not been validated in patient cohorts with an adequate sample size, so false-positive results associated with over-fitting of data and external factors other than IC that could influence the urine metabolome cannot be ruled out (39).

Distinct *Candida* metabolite signatures identified by GC-MS, LC-MS, ultra-performance LC-MS or hydrogen NMR (^1^H-NMR) spectroscopy were used to discriminate between (i) the most prevalent microorganisms responsible for bloodstream infections (candidemia and bacteremia) (175), (ii) *Candida* clinical isolates from different sources of infection (176), (iii) *Candida* species (based on their volatomes or volatile organic compound patterns) (177, 178), and (iv) *C. albicans* strains or *C. auris*/*C. auris* clades with distinct antifungal susceptibilities (175, 179, 180). Of the broad panel of metabolites identified, arabitol (or arabinitol) proved to be a useful biomarker candidate to distinguish between the most common causative agents of candidemia and bacteremia (in line with earlier studies (165, 166, 167, 168, 169, 170, 171)), as well as between *Candida* strains resistant and susceptible to antifungal drugs, such as azoles (fluconazole), polyenes (amphotericin B), and antimetabolites (5-flucytosine) (175, 179). Nevertheless, further research is needed to assess the clinical application of these metabolite signatures in the diagnosis of IC, reduce potential redundancies, and optimize the minimum number of biomarker candidates within metabolite signatures with the best accuracy. Because of the different antifungal susceptibilities of *Candida* species (29), it is reasonable to expect that the resulting validated and refined metabolite signatures may help clinicians not only to improve IC diagnosis, but also to select the appropriate antifungal treatment and decrease the selective pressure for antifungal resistance among *Candida* clinical isolates.

## Lipidomics

Lipidomics is a subfield of metabolomics that studies the lipidome (the entire complement of lipid species within a cell, organ, or biological fluid at a given time, location, and condition). Despite sharing similar experimental workflows and analytical techniques with metabolomics, this branch of metabolomics arose because these hydrophobic biomolecules exhibit greater structural complexity and molecular diversity than other metabolites (181, 182). In addition to being important structural components of cell membranes, metabolic precursors, regulators of cellular functions, signaling biomolecules, and energy sources in most organisms, lipids also play key roles in host-pathogen interaction, virulence, and antifungal resistance in many pathogenic fungi (183, 184). Indeed, lipidomics is emerging as a promising tool for understanding the pathophysiology of infections and discovering biomarkers for the diagnosis, prognosis, and therapeutic monitoring of infectious diseases (185, 186, 187). However, studies reporting the use of lipidomics for the diagnosis of IC are scarce and require further clinical evaluation (Fig. 1).

Two experimental studies examined the lipidomic and metabolomic patterns of liver tissues in a murine model of IC by FT-IR spectroscopy or LC-MS/MS (188, 189). These highlighted that IC was associated with an increase in lipid peroxidation and lipid/protein ratio, a reduction in lipid content and saturated/unsaturated lipid ratio, and an accumulation of conjugated bile acids (possibly due to liver damage and dysfunction caused by *C. albicans*). Similarly, serum lipidome profiles analyzed by ESI-MS could discriminate between IC and non-IC patients (190). These revealed two potential human lipid biomarkers for IC diagnosis, i.e., leukotriene F4 (LTF4) and 5,6-dihydroxy-eicosatetraenoic acid (5,6-diHETE), which exhibited lower serum levels in IC patients than in non-IC patients. Measurement of LTF4 and 5,6-diHETE in serum showed a specificity of 100%, but only a sensitivity of 58% and 33%, respectively, for IC detection. Since these eicosanoid lipids are involved in the regulation of inflammation and immune response, their lower abundance in IC could be related to the maintenance and progression of *Candida* infection (190, 191). Bearing these remarks in mind, future validation studies should focus on the detection of LTF4 and 5,6-diHETE alone and in combination for the diagnosis of IC, as well as for the prognosis and clinical follow-up of IC.

Several studies assessed the efficacy of *Candida* lipidomic profiling in the identification of *Candida* clinical isolates to the species level (177, 192, 193), as well as the detection of their antifungal susceptibilities (179, 194, 195, 196, 197). Based on their lipid patterns (at the *m/z* range of 600–1000), the handheld bipolar and high-throughput rapid evaporative ionization MS (REIMS) platforms proved to be a rapid and accurate method for species-level identification of 153 *Candida* clinical isolates (192). Different *Candida* lipid species, including phosphatidic acid (PA 36:4), phosphatidylserine (PS 40:4 and PS P-42:6), phosphatidylglycerol (PG 50:1) and hexosylceramide (HexCer d37:2) species, were identified as specific biomarker candidates for *C. albicans*, *C. parapsilosis*, *C. glabrata*/*N. glabrata*, *C. tropicalis*, and *C. krusei*/*P. kudriavzevii*, respectively. Like MALDI-TOF MS, rapid evaporative ionization MS has some disadvantages, such as their dependence on positive cultures (see above), high instrument cost, and spectral database limitations. Nonetheless, it does not require laborious sample pretreatment and extraction steps. Phospholipidomic profiling by ESI combined with tandem MS (MS/MS) revealed specific phospholipid molecular species for each *Candida* species, which allowed the detection of eight common etiologic agents of IC (193). On the other hand, distinct lipidome signatures and biomarker candidates identified by hydrophilic interaction LC-MS, GC-MS, targeted lipidomics (MRM), or stimulated Raman scattering were used to distinguish between *Candida* strains resistant and susceptible to amphotericin B or azoles (179, 194, 195, 196, 197). These antifungal drugs directly or indirectly affect *Candida* ergosterol (the major sterol lipid of fungal membranes), respectively. Amphotericin B extracts ergosterol from lipid bilayers forming large extra membranous aggregates, whereas azoles inhibit its biosynthesis (198). High content of glyceryl phosphatide fatty acids (with an odd number of C atoms and more than 34-C), phosphatidylcholines (PC), phosphatidyl ethanolamines (PE) and phosphatidylglycerols, low lysoPC, lysoPE, and lysoPG content, as well as aberrant lipogenesis were proposed as potential lipidomic biomarkers of azole-resistant *C. albicans* (179, 196). In contrast, higher unsaturation index and elevated free ergosterol levels were associated with azole-resistant strains of *C. auris*/*C. auris* (195). Distinct sphingolipid species signatures revealed by MRM were able to differentiate between amphotericin B and fluconazole-resistant *C. auris*/*C. auris* isolates, and uncovered glucosylceramide (GlcCer) with d19:2 GlcCer backbone as a sphingolipidomic biomarker candidate of amphotericin B-resistant *C. auris*/*C. auris* strains (197). Despite their encouraging results, all these lipidomics-based assays remain investigational. More research is therefore necessary to elucidate whether they could replace traditional methods of species-specific identification and antifungal susceptibility testing in patients at risk for IC in routine clinical practice.

## Glycomics

Because of the uniqueness, complexity, and functional specificity of glycans compared to other metabolites, glycomics has also emerged from metabolomics to investigate the glycome (the complete repertoire of glycan structures in a biological system at a specific time, location, and condition). Glycans are complex sugar molecules (oligosaccharides and polysaccharides) that can be found in free form or covalently bound to proteins (glycoproteome) and lipids (glycolipidome) (199, 200). Developments in analytical techniques, such as hydrophilic-interaction, reverse-phase or porous graphitized carbon LC-MS, capillary electrophoresis (CE)-MS, ion mobility spectrometry-MS, NMR spectroscopy, and glycan, lectin or anti-glycan antibody microarrays, among others, have improved the characterization of the glycome in various physiological and pathological processes (201, 202, 203). Since glycans are important components of host and microbial cell surfaces that mediate host-pathogen interactions (recognition, adhesion, colonization, invasion, immune response, and immune evasion), there has been growing interest in their study to gain new insight into the infectious process and to identify clinical biomarkers and signatures of infectious diseases, including IC (Fig. 1) (204, 205, 206, 207).

Two *Candida* glycan biomarkers discovered in the pre-omics era to diagnose IC, i.e., (1, 3)-β-D-glucan (BDG) and mannan, remain part of routine testing in many clinical mycology laboratories (206, 208, 209, 210). These glycan biomarkers are the most abundant polysaccharides of the cell wall of *Candida* and other pathogenic fungi (211, 212). There are several commercial assays for their measurement in clinical samples (serum, plasma and/or urine) from IC patients (15, 213). The BDG test has a high negative predictive value and is widely used in daily clinical practice to rule out IC (214, 215). However, this nonculture-based assay is prone to false-positive results because of several possible sources of contamination (such as glucan-containing materials, hemodialysis, human blood products, some antibiotics, and severe mucositis, among others), and the panfungal nature of BDG (which does not discriminate IC from other fungal infections, and requires species-specific identification) (15, 216). Unlike the BDG test, the (nonculture-based) mannan assay is associated with false-negative results due to the rapid clearance of *Candida* mannan from the bloodstream and the formation of immune complexes (15, 166). Nevertheless, the diagnostic accuracy of the BDG and mannan tests can be improved by their combined use, allowing clinicians to guide pre-emptive therapeutic decisions in patients at risk for IC (214, 217).

In a recent study, a *Candida* glycan signature detected by MALDI-TOF MS in serum samples was able to differentiate between IC and non-IC patients, and uncovered a disaccharide biomarker for IC diagnosis (218). This biomarker candidate was then identified as trehalose, which can be released from *Candida* cells into the bloodstream during the infectious process under different stress conditions (219, 220). This glycan signature was used to create the “MS-disaccharide (MS-DS) index” for IC detection (221, 222). This MS-DS index was calculated as the ratio of *m/z* 365 (trehalose) to *m/z* 361 (matrix) signal intensities (221), and was further validated in a multicenter clinical trial (222). Like BDG, the MS-DS index proved to be a panfungal biomarker that requires species-specific identification and can complement the high specificity of the *Candida* mannan assay for early and cost-effective diagnosis of IC (221, 222).

## Immunomics

The immunome is the complete roadmap of the immune responses of a given host interacting with a foreign antigen, self-antigen, foreign allergen, or their epitopes (223, 224). Therefore, knowledge of its variation is crucial for better understanding host-pathogen interactions and immune responses, as well as for detecting clinical biomarkers and targets involved in infectious diseases, cancers, autoimmune diseases, and allergies for their potential inclusion in future diagnostic, vaccine, or immunotherapeutic applications (225, 226). Immunomics is a relatively new and expanding scientific field that combines immunology with omics technologies, bioinformatics, and clinical medicine, among others, to study the immunome of a biological system at a given time, location, and condition. Several approaches based on the host immune response directed against the immunome of *Candida*, i.e., the subset of its proteome (immunoproteome), peptidome (immunopeptidome), and glycome (immunoglycome) that interface with the immune system, before, during, or after IC have been developed over the past few decades. These have been successfully applied to unravel the complexity of the interaction between *Candida* and the host immune system, as well as to identify potential immunomic biomarkers for IC diagnosis, prognosis and follow-up, as well as promising targets for IC vaccines and immunotherapeutics, as detailed below (Fig. 1).

### Immunoproteomics

Various immunoassays based on enzyme-linked immunosorbent assay (ELISA), immunofluorescence, latex agglutination, or lateral flow, to name but a few, have evaluated the potential of serum antibodies against one or several immunogenic *Candida* proteins for the diagnosis of IC (38, 227, 228, 229, 230, 231). Among them, the *C. albicans* germ tube antibody (CAGTA) assay, which targets a panel of immunogenic *Candida* proteins (232), was associated with high specificity but moderate sensitivity for IC detection (206, 233). The CAGTA assay added diagnostic value to the BDG or mannan tests (see above), so their combined use for IC diagnosis can be recommended according to the current ESCMID guidelines (206, 214, 234, 235). However, these immunoassays are prone to provide false-positive results in healthy subjects (owing to the commensal nature of some *Candida* species) and false-negative results in immunocompromised patients (due to their weak mounted antibody response) (15, 166). Different immunoproteomic approaches based on forward or reverse proteomics (predicted or cloned open reading frames (ORFs) of an organism, respectively (236, 237)) have been designed to overcome the limitations of these classical immunoassays, profile the *Candida* immunoproteome in IC, and elucidate its potential as a mine for development of clinical biomarkers and vaccines for IC (238, 239, 240, 241, 242).

*In vivo* induced antigen technology (IVIAT) represents a powerful approach to identify microbial genes encoding immunogenic proteins that are expressed *in vivo* during the infectious process (243, 244). Serum antibody-based profiling of a *C. albicans* genomic DNA expression library by IVIAT uncovered 10 *C. albicans* immunogenic protein-coding genes (including virulence determinants, such as *CST20*, *CPP1*, *HWP1*, *NOT5*, and *RBF1*) that were associated with oropharyngeal candidiasis (245, 246). Although their potential role in IC diagnosis needs to be further examined, IVIAT has the advantage of identifying less abundant gene products, but the disadvantage of not detecting their possible posttranslational modifications (PTMs), among others (246).

Serological analysis of recombinant complementary DNA (cDNA) expression libraries (SEREX) is one of the oldest immunoscreening strategies used to reveal disease-specific antigens that can be exploited for the development of diagnostics and therapeutics in infectious diseases, cancers, autoimmune diseases, and allergies (247, 248, 249, 250). Like IVIAT, SEREX is highly sensitive but lacks the ability to detect PTMs (246). Different SEREX studies identified several complementary DNA clones coding immunogenic *C. albicans* proteins (such as *HSP70*, *ENO1*, *PRA1*, *HYR1*, and *APE2*, among others) that could serve as diagnostic targets for IC (238, 239, 251, 252, 253, 254). Nonetheless, these require further clinical evaluation in larger cohorts of patients at risk for IC.

Serological proteome analysis (SERPA) is a top-down immunoproteomic approach based on the combination of two-dimensional gel electrophoresis with western blotting and MS (255, 256, 257). This has been widely used to globally profile the antibody response to the secretome (258, 259), cell wall proteome (260, 261), and intracellular proteome (262, 263, 264) of *C. albicans* in IC patients. SERPA identified a broad panel of serologic biomarker candidates for diagnosis (258, 259, 260, 263, 264, 265), prognosis (260, 266), and follow-up (263, 267) of IC, as well as potential targets for immunotherapeutics and vaccines against IC (260, 268). Among these biomarkers, elevated serum levels of anti-*Candida* Bgl2 antibodies were an independent predictor of IC and provided indirect protection against IC (260). SERPA also revealed a serum antibody signature directed against *Candida* Eno1 and Hsp90 that discriminated between IC and non-IC patients (264), as well as another against *Candida* Hsp90, Met6, Pgk1, Ssb1, and Tdh3 that differentiated between IC patients with good and poor clinical outcomes at presentation (266). Several of these biomarker candidates and signatures were validated in appropriate single and multiplexed prototype immunoassays (227, 228, 229, 230, 231, 240, 264, 266, 269), supporting their clinical utility for the diagnosis and prognosis of IC. However, larger-scale evaluations are still required before their potential translation to clinical practice. Furthermore, SERPA was useful for characterizing speciation of the *Candida* immunoproteome and identifying multiple serologic biomarker candidates at the chemical and molecular level in IC (270, 271, 272). In particular, SERPA highlighted that immune recognition of two discrete antigenic species of Eno1 and Pgk1, or glycosylated species of Sap6, was specifically associated with IC (258, 270). A better understanding of their potential distinctive modifications (such as amino acid substitutions or PTMs) may provide the basis for the rational design and molecular optimization of future diagnostic and prognostic kits, vaccines, and immunotherapies for IC and other infectious diseases (270).

Immunoaffinity MS (IAMS) is a complementary approach for rapid and cost-effective serological testing of the antibody response to the immunome of a (micro)organism (273, 274). It combines immunoaffinity-based enrichments of disease-associated antigens that elicit a specific immune response with their identification and quantification by MS. This immunoproteomic method proved to be a promising tool for identifying immunogenic proteins from the hyphal secretome and surfome of *C. albicans* (including Als1, Als3, Hwp1, and Ssa2, among others) that triggered a specific IgA antibody response in the murine gut (275). However, its usefulness for the diagnosis of IC requires further investigation.

Antigen microarrays are a high-throughput method for the multiplexed measurement of serum antibodies against panels of multiple antigenic proteins (276). Two *C. albicans* antigenic protein microarrays were able to discriminate between IC and non-IC patients (241, 242). In addition, these immunoassays unveiled a broad set of biomarker candidates for (i) IC diagnosis (antibodies against *Candida* Bgl2, Eno1, Grp2, and Pgk1 (241), and 13 *Candida* cell surface antigens associated with drug resistance and oxidative stress (242)), and (ii) clinical follow-up of IC (antibodies against 33 *Candida* cell surface antigens related to heme-associated iron acquisition (242)). These microarray assays remain investigational and need further standardization and validation before clinical application can be achieved.

### Immunopeptidomics

B-cell epitope mapping using arrays of overlapping synthetic peptides derived from *C. albicans* Pra1 (mp58/Fbp1) or Sap2, or using single epitopes selected from *C. albicans* Hsp90 identified several antibody-binding sites (continuous or linear B-cell epitopes) on these antigen proteins that were associated with IC (277, 278, 279). Profiling of *C. albicans*-specific human T-cell clones proved to be a useful tool to characterize the major histocompatibility complex (MHC) class II-bound peptides (T-helper cell epitopes) of *C. albicans* Mp65, as well as their processing requirements (280). Nevertheless, the clinical relevance of these immunopeptide biomarker candidates for IC serodiagnosis and their potential as vaccine candidates should be evaluated.

Immunoinformatic analyses based on 3D modeling, reverse translation, and codon optimization on different immunodominant B-cell epitopes in-silico predicted from 8 *C. albicans* antigens (Als, Bgl2, Eap1, Ece1, Hyr1, Hwp1, Met6, and Sap1) and connected with a linker revealed that this construct could accurately discriminate IC patients from non-IC patients (281). Likewise, immunoinformatic analysis based on in-silico prediction identified the immunogenic T-cell epitopes of *C. albicans* Sap2 that bind to the HLA-DRB1 supertype (282). Further experimental studies are needed to confirm the results from these immunoinformatics-based approaches and elucidate whether they could provide the basis for the rational design of new-generation diagnostics and vaccines for IC.

### Immunoglycomics

The first anti-glycan biomarker discovered to detect IC was the anti-*Candida* mannan antibody (283). Several studies have shown that its limited diagnostic value for the diagnosis of IC can be improved by its combined use with the mannan test (see above) (284, 285, 286). In fact, this combination and serial determinations of both assays in serum or plasma are recommended by the current ESCMID guidelines (214). Similarly, antibodies against other glycans (glucan and chitin) of the *Candida* cell wall were also proposed as biomarker candidates for IC detection that complement the mannan test (287).

xMAP and surface plasmon resonance analyses using Luminex and Biacore technologies, respectively, assessed the recognition of α- and β-mannan epitopes (α- and β-oligomannosides) by anti-*Candida* mannan antibodies using synthetic biotin sulfone-tagged oligomannosides (288, 289). These analyses revealed that serum antibodies directed against α-1,2-mannobioside (2α-Man), β-1,2-mannobioside (2β-Man) and β-1,2-mannotriose (3β-Man) were associated with IC (289). Furthermore, 2β-Man to 2α-Man recognition ratio was able to discriminate both between IC and non-IC patients at ICU, and between IC patients and healthy individuals. These promising results need to be confirmed in other high-risk populations to guide the success of their clinical translation in the future.

## Imiomics

Imaging-omics, also coined as imiomics, has recently emerged to analyze and map pointwise correlations between whole-body image data (local physical properties at each body point) and nonimaging data (global measurements of nonimaging biomarkers) in large cohorts of patients with a high degree of spatial resolution (290, 291, 292). In contrast to other classical methods based on predefined regions of interest, imiomics captures intra-organ and intratissue variations. It is therefore a valuable tool for better understanding the biology and pathogenesis of diseases, and minimizing the number of misdiagnoses. Nevertheless, its application to detect infectious diseases is still in its infancy.

In a preclinical whole-body imaging study of IC, antibody-directed positron emission tomography and magnetic resonance imaging (immunoPET/MRI) in a murine model of IC accurately and noninvasively detected deep-seated candidiasis in different organs (kidney, brain, spleen, muscle, and colon) of *C. albicans*-infected mice (293). Molecular imaging was guided by a disease-specific tracer based on immunodetection of a putative β-1,2-mannan epitope present in the cell wall of *C. albicans*. In light of the promising results of this preclinical study, when traditional diagnostic methods fail to identify IC (in particular, blood culture-negative, deep-seated candidiasis (11)), clinicians could turn to imiomics to aid in diagnosis, without the need for deep tissue biopsy sampling (contraindicated in patients with severe thrombocytopenia or coagulopathies, and in critically ill or unstable patients (14, 18)).

## Microbiomics and mycobiomics

There is increasing evidence that the human microbiome affects health and disease (294). The human microbiome is the entire set of microbial communities inhabiting the human body, including their microorganisms (bacteria, archaea, fungi, parasites, and viruses), genomes and products (genes, proteins, and metabolites), and ecosystem (295, 296). Because of its important role in health and disease, microbiomics is gaining attention as a screening tool to study the dynamics, function, and composition of the human microbiome, and to evaluate its use for the diagnosis, prevention, and treatment of diseases, including *Candida* infections (297, 298, 299). This ever-expanding field of research combines environmental or clinical metadata with metagenomics, metatranscriptomics, metaproteomics, and metabonomics, among others (Fig. 1) (295, 300, 301). However, these methods are often associated with a lack of standardization for their application in routine clinical practice (302, 303).

Metagenomic NGS was a powerful alternative method for early identification of *Candida* spp. in IC patients with negative culture or PCR results (304, 305, 306, 307), or coinfected with other pathogens (308, 309). Nanopore-based metagenomic sequencing was able to identify *Candida* species in IC patients in a higher detection rate and shorter turnaround time than cultures and other conventional diagnostic methods (310, 311). Shotgun metagenomic, metatranscriptomic, and metaproteomic analyses of fecal samples from a premature infant with IC caused by *C. parapsilosis* showed significant changes in the gene expression and proteome patterns of bacteria and fungi (312). These included co-shifts in the relative abundance of *C. parapsilosis* and *Enterococcus faecalis* over time. High-resolution analysis of the mycobiome (the fungal component of the microbiome) of patients with candidemia revealed a substantial intestinal expansion of pathogenic *Candida* spp. and their subsequent translocation to the bloodstream prior to the development of IC (313). This was associated with a reduction in the burden and diversity of bacterial species (mainly anaerobic bacteria). Metagenomic analyses in animal models with IC caused by *C. albicans* revealed gut microbiota signatures associated with IC (314, 315). Metabonomic analysis by LC-MS further showed that fecal metabolic profiles discriminated between candidemia and control mice (315). Although these findings support microbiomics-based assays to guide pre-emptive therapeutic strategies in patients at risk for IC, further standardization and validation studies on these dynamic changes in the microbiome of IC patients are needed for future use in diagnostic applications.

## Multiomics and Panomics: Toward Next-Generation Diagnostics and Precision Medicine

**Fig. 2:**
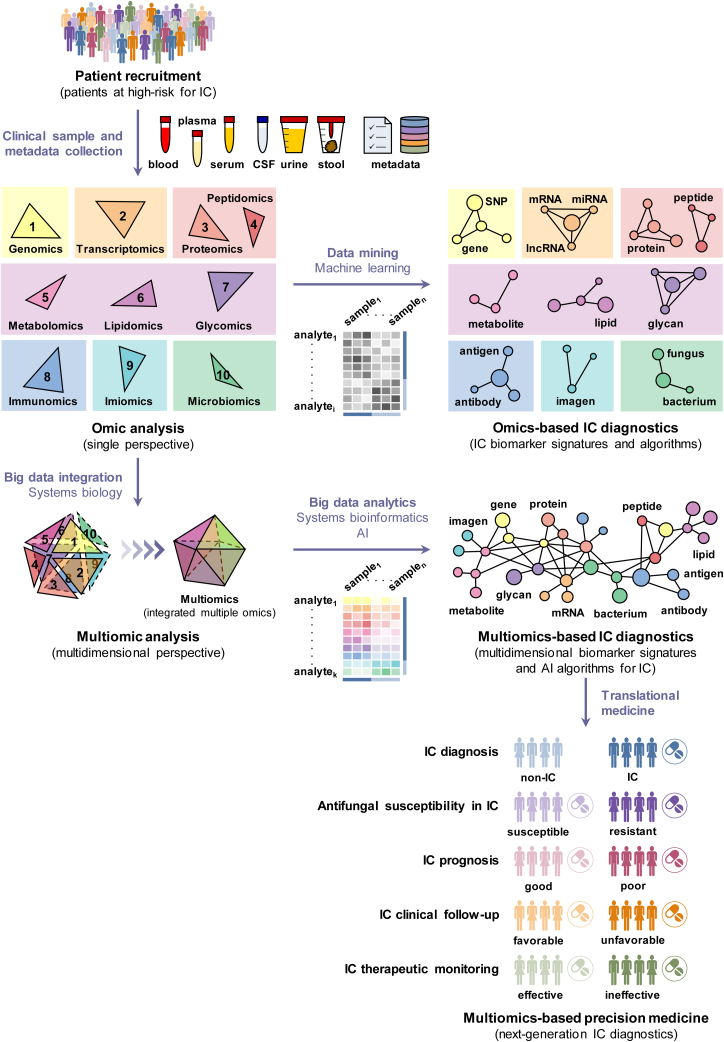
**Steps toward next-generation IC diagnostics for precision medicine.** The integration of multiple levels of molecular information (*n-D polytopes* versus *2-D polytopes*) into multidimensional biomarker signatures and computational algorithms using multiomics and AI is crucial for achieving a comprehensive understanding of the pathophysiological mechanisms underlying IC, and opening the door of precision medicine in IC. See text for further details. AI, artificial intelligence; CSF, cerebrospinal fluid; IC, invasive candidiasis; lncRNA, long noncoding RNA; miRNA, microRNA; SNP, single nucleotide polymorphism.

Over the past few decades, omics technologies have accelerated the discovery of biomarker candidates for early diagnosis, prognosis, follow-up, and therapeutic monitoring of IC (as detailed above). Given the complex, heterogeneous, multifaceted, and multisystem nature of IC pathogenesis (316, 317), it is conceivable that no single omics strategy will suffice to help clinicians make more precise and personalized clinical decisions in IC patients. The integration of multiple omic datasets offers an unprecedented opportunity to fully understand the molecular mechanisms of disease pathogenesis across multiple levels of biology, better describe geno-pheno-envirotype associations, and translate this multilevel information into next-generation diagnostics for future personalized interventions (318, 319, 320). Multiomics or, better yet, panomics (the integration of multiple or all omics technologies, respectively) together with systems biology and artificial intelligence (AI) are expected to uncover a combination of multiple and more informative clinical biomarkers at different molecular levels of complexity for comprehensive disease diagnosis in a precise and personalized approach (321, 322, 323) (Fig. 2). However, their application to the diagnosis of IC is still in its infancy, so their usefulness and translation to routine clinical practice will have to be further evaluated in the future.

Only a few multiomics-based studies have recently focused on the diagnosis of IC (123, 312, 324, 325, 326, 327). These studies combined (i) genomics and transcriptomics for the detection of persistent candidemia (123), (ii) metagenomics, metatranscriptomics, and metaproteomics for the monitoring of candidemia caused by *C. parapsilosis* in a premature infant (312), (iii) metagenomics, transcriptomics, and metabonomics for the identification of traits indicative of the transition from gastrointestinal colonization of *C. albicans* to translocated IC in animal models (324), (iv) genomics and transcriptomics for the detection of multidrug resistance profiles of *Candida haemulonii*/*Candidozyma haemuli* strains isolated from patients with candidemia (21, 325), (v) genomics, transcriptomics, and phenomics for the identification of pan-drug resistance patterns of *C. auris*/*C. auris* clinical isolates from an IC patient (326), and (vi) proteomics, metabolomics, and lipidomics for antifungal susceptibility testing of *C. auris*/*C. auris* strains isolated from IC patients (327). Genomic and transcriptomic profiles of multidrug-resistant and multidrug-susceptible *Candida* isolates pointed to a multifactorial mechanism of multidrug resistance (325, 327), supporting the importance of integrating multiple omic data to achieve precision medicine.

Several algorithms and mathematical models based on the application of machine learning, a branch of AI (328), to single omics datasets have been developed and validated for the prediction of IC risk, antifungal resistance in *Candida* clinical isolates, or clinical outcomes in IC patients at presentation (117, 118, 119, 139, 264, 266, 270, 329). In light of these valuable data, AI should be implemented in future multiomic or panomic data integration studies to reveal multidimensional biomarker signatures of diagnosis, antifungal susceptibility, prognosis, follow-up, and therapeutic monitoring for IC that improve predictive accuracy in precision medicine beyond those provided by single omic approaches (Fig. 2). Although there are currently several significant challenges to their clinical implementation (330, 331, 332), these next-generation IC diagnostics promise to revolutionize medical practice by helping clinicians make diagnostic and therapeutic decisions based on precision medicine. Ultimately, these diagnostics that integrate multilevel information could enable the application of early, personalized, targeted therapies (rather than traditional one-size-fits-all approaches) that improve the clinical outcomes of IC patients by taking into account their interindividual differences.

## Conclusions and Future Prospects

The 21st century has witnessed the discovery of an increasing number of biomarker candidates for IC diagnostics driven by different omics (genomics, transcriptomics, proteomics, peptidomics, metabolomics, lipidomics, glycomics, immunomics, imiomics, and microbiomics) and multiomics technologies. Although some omics-based IC diagnostic methods are already used in routine clinical practice, most of them are still in the research phase and none of them has proven to be perfect for diagnosing IC and minimizing its high morbidity and mortality rates and health care costs. Therefore, in the coming years, it will be necessary to bridge the gap between discovery research and translation into the clinics, and to solve its main underlying challenges. Future research and development efforts should also aim at (i) investigating the contribution of other emerging omics technologies (such as epigenomics, epitranscriptomics, metapeptidomics, metabolipidomics, metaglycomics, and metaimmunomics, among others) to the process of biomarker development for IC diagnosis, in line with previous studies (333, 334, 335, 336), (ii) integrating multiomic or panomic data, AI, and patient metadata for the discovery of multidimensional biomarker signatures and computational algorithms for IC diagnosis, (iii) standardizing, validating, and refining these multidimensional signatures and AI-based algorithms in multicenter studies with larger prospective cohorts of IC patients, (iv) evaluating their diagnostic performance in IC (especially, deep-seated candidiasis in the absence of candidemia) in low and high prevalence settings, and (v) promoting their translation into POC testing devices that can help guide more precise and personalized clinical decisions (i.e., multiomics- or panomics-based precision medicine) at the bedside. Hopefully, these future efforts could be the key to precision medicine in IC.

## Conflict of Interest

The authors declare no competing interests.
